# Characterization of KRAS Mutational Regression in Oligometastatic Patients

**DOI:** 10.3389/fimmu.2022.898561

**Published:** 2022-07-22

**Authors:** Alessandro Ottaiano, Roberta Penta de Vera d’Aragona, Anna Maria Trotta, Mariachiara Santorsola, Maria Napolitano, Giosuè Scognamiglio, Fabiana Tatangelo, Paolo Grieco, Silvia Zappavigna, Vincenza Granata, Francesco Perri, Amalia Luce, Giovanni Savarese, Monica Ianniello, Marika Casillo, Nadia Petrillo, Andrea Belli, Francesco Izzo, Guglielmo Nasti, Michele Caraglia, Stefania Scala

**Affiliations:** ^1^ Istituto Nazionale Tumori di Napoli, Istituto di Ricovero e Cura a Carattere Scientifico (IRCCS) “G. Pascale”, Naples, Italy; ^2^ Oncohaematology Department, Azienda Ospedaliera di Rilievo Nazionale (A.O.R.N.) Santobono-Pausilipon di Napoli, Naples, Italy; ^3^ Department of Pharmacy, University of Naples “Federico II”, Naples, Italy; ^4^ Department of Precision Medicine, University of Campania “L. Vanvitelli”, Naples, Italy; ^5^ Biogem Scarl, Institute of Genetic Research, Laboratory of Molecular and Precision Oncology, Ariano Irpino, Italy; ^6^ AMES, Centro Polidiagnostico Strumentale srl, Naples, Italy

**Keywords:** oligo-metastatic disease, colorectal cancer, KRAS, cytotoxicity, HLA

## Abstract

**Background:**

We previously reported rare regressive genetic trajectories of *KRAS* pathogenic mutations as a specific hallmark of the genuine oligometastatic status in colorectal cancer (CRC).

**Methods:**

Survival and prognostic impact of disease extent in 140 metastatic CRC patients were evaluated through the Kaplan–Meyer curves and the Log-Rank test. *KRAS* mutations were assessed through the Illumina NovaSeq 6000 platform and TruSight™ Oncology 500 kit. HLA typing was carried out by PCR with sequence-specific oligonucleotides. Lymphocyte densities in tumors were expressed as cells per square millimeter. NKs isolated and CD8^+^ from NK-depleted PBMCs were characterized through flow cytometry. CD107a externalization was evaluated as NKs/CD8 cytotoxicity toward human colon cancer cells HT29, SW620, HCT116, and LS174T carrying different *KRAS* mutations.

**Results:**

The oligometastatic status was a strong and independent variable for survival (HR: 0.08 vs. polymetastatic disease; 95% CI: 0.02–0.26; *p* < 0.001). Eighteen oligometastatic patients were selected. Twelve were alive at the last follow-up, and 9 were characterized. Genetic regression of *KRAS* was observed in 3 patients: patient (PAT)2, PAT5, and PAT8. PAT2 and PAT5 presented the highest levels of GrzB^+^ lymphocytes in the tumor cores of the metastases (120 ± 11.2 and 132 ± 12.2 cells/mm^2^, respectively). Six out of 9 patients (67%), including PAT2 and PAT5, expressed HLA-C7. Twopatients (PAT2 and PAT5) presented high CD3^+^/CD8^+^-dependent cytotoxicity against HLA-C7+ SW620 cells (p.G12V-mutated cells), which was consistent with their observed mutational regression (p.G12V/p.G13D in primary→p.G13D in metastatic tumor).

**Conclusions:**

We provide evidence that CD3^+^/CD8^+^ lymphocytes from oligometastatic CRC patients display differential cytotoxicity against human colon cancer cells carrying *KRAS* mutations. This could provide an interesting basis for monitoring oligometastatic disease and developing future adoptive immunotherapies.

## Introduction

Colorectal cancer (CRC) is a dismal disease and is the second cause of cancer-specific death worldwide. Although the introduction of biological drugs (bevacizumab, panitumumab, cetuximab, aflibercept, and regorafenib) in the treatment of metastatic CRC (mCRC) has improved the prognosis, the 5-year survival rate is not higher than 5% (1).

We have previously reported that the presence of regressive genetic trajectories in key driver genes is associated with the oligometastatic status of a low-burden disease and a clinical indolent course (2). In this light, we described the loss of *KRAS* pathogenic mutations from primary to metastatic tumors in a well-defined model of lung-specific oligometastatic disease, allowing a clean genotype/phenotype correlation study (3). Similar genetic changes were found in a highly selected model of patients with liver metastases from CRC compared to metastatic lesions (4). However, we could not exclude the hypotheses of either “back” (or regressive) mutation or immune-mediated recognition of neoplastic clones bearing specific pathogenic mutations (immune-mediated subclonal selection) responsible for *KRAS* regression. This biological effect is highly relevant to understanding the biology of CRC and can create new scenarios in the therapeutic strategies for the disease.

Interestingly, we noted that patients with *KRAS* regressive mutations also presented a higher infiltration of CD8^+^-activated T cells. CRC neoplastic masses are infiltrated by different leucocyte subsets (macrophages, eosinophils, NKs, CD8^+^ T cells, etc.) that dynamically interact with tumor cells within the tumor microenvironment (TME). The final result of this clash is largely undefined and, in many cases, can prompt neoplastic progression depending on the predominant TME-infiltrating subsets (5–7). Recent robust evidence suggests that both NKs and CD8^+^ T cells (tumor-infiltrating lymphocytes (TILs)) are associated with better prognosis and are able to “sculpture” the neoplastic population by eliminating some neoplastic clones, including those carrying KRAS mutations (8–11).

In the present study, taking advantage of the opportunity to study alive patients representing a well-defined and characterized model of oligometastatic disease, we have explored their immunologic characteristics, including HLA haplotypes, tumor immune microenvironment, and reactivity of peripheral lymphocytes against the differentially KRAS-mutated colon cancer cells.

## Methods

### Definition of Oligometastatic Disease and Patients’ Management

Consecutive metastatic colorectal cancer (mCRC) patients treated at the Department of Abdominal Oncology, Substructure of Innovative Therapies for Abdominal Cancers, Istituto Nazionale Tumori (National Cancer Institute), “G. Pascale” Foundation, were analyzed. Oligometastatic patients were intended as those having one to three lesions per organ with a maximum tumor diameter smaller than 70 mm and no lesions encompassing 25 mm in diameter. Otherwise, patients were considered polymetastatic. Data were extracted from an institutional electronic database prospectively updated (January 2018–December 2021). To avoid clear negative prognostic influences, patients with an Eastern Cooperative Oncology Group (ECOG) performance status score of >1, age >80 years, life expectancy of <3 months, and BRAF-mutated tumors were excluded.

Treatments and follow-up procedures were applied according to ESMO (European Society of Medical Oncology) guidelines (12). Authorization from the Scientific Directorate (Prot. 03b-2020 TIMA) in the context of an ongoing retrospective no-profit study on long-term survivors in CRC (sponsored by the Lega Italiana per la Lotta Contro I Tumori-Naples section) for recovering additional blood from oligometastatic patients was obtained. All patients signed an informed consent specifically referring to this project.

### NK and NK-Depleted PBMC Preparation

Peripheral blood mononuclear cells (PBMCs) from oligometastatic CRC patients were freshly isolated using Ficoll Paque (GE Healthcare, Uppsala, Sweden) gradient centrifugation. CD3^−^CD56^+^ NK cells were isolated from PBMCs by negative selection using magnetic beads (NK Cell Isolation Kit, human, San Diego, CA 92121, USA, Miltenyi Biotec). The noneluted fraction was recovered and used as NK-depleted PBMCs. NK and NK-depleted PBMCs were stimulated with recombinant interleukin-2 (rIL-2, 100 U/ml) for 18 h in RPMI 1640 medium (Cytiva HyClone™).

### Flow-Cytometric Analysis and Antibodies

Flow cytometry was performed on venous peripheral blood collected in heparin-coated vacutainer tubes using a FACSAria III 8-colour flow-cytometer (BD Biosciences, San Jose, CA, USA), daily calibrated with caliBRITE beads (Fitc, Pe, PerCP, and APC) and compbeads (Pe-Cy7 and APC-Cy7; BD Bioscience, San Jose, CA, USA). Fluorochrome-labeled monoclonal antibodies (BD Bioscience) for the identification of NK and T cells were Horizon-V450-anti-CD16, fluorescent isothiocyanate (FITC)-anti-CD8, Pe-Cy7-anti-CD56, and APC-Cy7-anti-CD3. Viability was analyzed using LIVE/DEAD cell stain (Invitrogen). Cells were stained with antibodies for 30 min at 4°C and washed with FACS buffer (PBS/0.2% BSA/0.01% NaN_3_). A minimum of 100,000 events for each sample were collected and analyzed using FACSDiva™ 8.0 Software (BD Bioscience).

### Degranulation Assays

Target CRC cell lines were cocultured with rIL-2-activated NK and NK-depleted PMBCs at an E:T ratio of 10:1 in a total volume of 250 μl in 96-well flat-bottom plates in the presence of PE-anti-CD107a antibody at 37°C and 5% CO_2_. To detect spontaneous degranulation, effector cells were incubated in the absence of target cells; a positive control was effector cells in the presence of PMA (2.5 µg/ml) and ionomycin (0.5 µg/ml). Following a 3-h culture, cells were stained to detect degranulation in specific lymphocyte subpopulations (see *Antibodies*). CD107a externalization was analyzed on CD3^−^/CD56^+^ NK cells as well as CD3^+^/CD8^+^ from NK-depleted PBMCs. Normalized CD107a was the percentage of CD107a^+^ cells subtracted from spontaneous degranulation/PMA-IONO-induced (positive control) degranulation. All experiments were conducted in triplicate, and representative results are shown.

### CRC Cancer Cell Lines

The cytotoxic activity of peripheral T and NK cells was tested against human CRC cell lines: HT29 (*KRAS* wild-type), HCT116 (*KRAS* p.G13D), SW620 (*KRAS* p.G12V), and LS174T (*KRAS* p.G12D). All cells were cultured in the recommended growth medium supplemented with 10% heat-inactivated fetal bovine serum (FBS), 1% l-glutamine, and 1% penicillin/streptomycin and maintained in 95% air, 5% CO (2) at 37°C. Cell line identities were confirmed by short tandem repeat DNA typing at ATCC (VA, USA).

### *KRAS* Mutational Assessment

*KRAS* mutations were assessed on formalin-fixed paraffin-embedded (FFPE) primary and metastatic tumor tissues from oligometastatic patients. The DNA was extracted from three 10-µm FFPE sections through the MGF03-Genomic DNA FFPE One-Step Kit, according to the manufacturer’s protocol (MagCore Diatech). DNA quality was established in triplicate using the FFPE QC Kit according to the manufacturer’s protocol (Illumina, San Diego, USA). The libraries were prepared with TruSigt TMOncology 500 kit. Sequencing was performed on an Illumina NovaSeq 6000 (San Diego, USA) platform. The assay detects small nucleotide variants (SNVs), indels, splice variants, and immunotherapy biomarkers in 523 cancer-relevant genes. However, our analysis focused on *KRAS*-related genetic results. The Illumina TruSighth Oncology 500 bioinformatics pipeline was applied to analyze quantitatively and qualitatively the sequencing results, as previously reported (3, 4).

### Tumor Microenvironment Characterization

Analysis of lymphocyte subsets infiltrating TME was performed through immunohistochemistry (IHC). Formalin-fixed, paraffin-embedded 4-μm tissue sections of primary tissues and metastases were immunostained according to a biotin-streptavidin-peroxidase method (YLEM kit, Rome, Italy) (13). Treatment with primary antibodies consisted of anti-human CD3, anti-human CD8, anti-human FoxP3, and anti-human granzyme B. Slides were counterstained with hematoxylin, dehydrated, and mounted in Diatex. Negative controls were obtained by substituting the specific primary antibodies with a mouse myeloma protein of the same subclass (at the same concentration as the monoclonal antibody). Cell subsets infiltrating tumor cores (TC) and invasive margins (IM) (14) were counted by two pathologists six times and reported as cells per square millimeter (density).

### HLA Typing

Genomic DNA was extracted from fresh whole blood using a QIAmp DNA Blood Mini Kit (Qiagen, Manchester, UK). The HLA typing was carried out by PCR sequence‐specific oligonucleotide (SSO) using LABType^®^ SSO (One Lambda Inc., Los Angeles, CA, USA). Unresolved typing was investigated by sequence-based typing (SBT) analysis. Sequence alignments were done with alleles from the IMGT/HLA Sequence Database release 3.14.0 (14).

### Data Reporting and Analysis

The analysis of functional experiments and phenotypical characterizations are predominantly descriptive. Experiments were performed in triplicate; they gave homogeneous results. Overall survival (OS) of the patients was measured from the start of the first-line chemotherapy until death from any cause. Survival was shown through the Kaplan–Meier curves. Differences in survival according to the extent of disease (oligo- vs. polymetastatic disease) before starting the first-line treatment were evaluated through the Log-Rank test. A Cox proportional hazards regression model was used to analyze the effect of potential factors (covariates) influencing OS. Dichotomized covariates were age, gender, side of the primary tumor, response to first-line therapy, RAS gene status, and oligometastatic disease. Hazard ratios (HR) were intended to measure the risk of death, at any time, for a patient having a specific risk factor present compared with a patient with that risk factor absent, given both patients are the same on all other covariates. The 95% confidence intervals (CI) of HR were also reported. All statistical analyses were performed using the MedCalc^®^ 9.3.7.0 and Excel software. *p* < 0.05 were considered statistically significant. According to the internal policies of our Institute, the institutional review board approval was not required for the retrospective analysis of this clinical cohort. The lymphocyte densities in tumor tissues were reported with the arithmetic mean of ±2 standard deviations (SDs). Standard “box-and-whisker” graphs were used to plot cell densities of means from different patients in a comparative perspective (primary vs. metastatic tumors).

## Results

### Clinical Context and Selection of Good-Prognosis Oligometastatic CRC Patients

**Figure 1:**
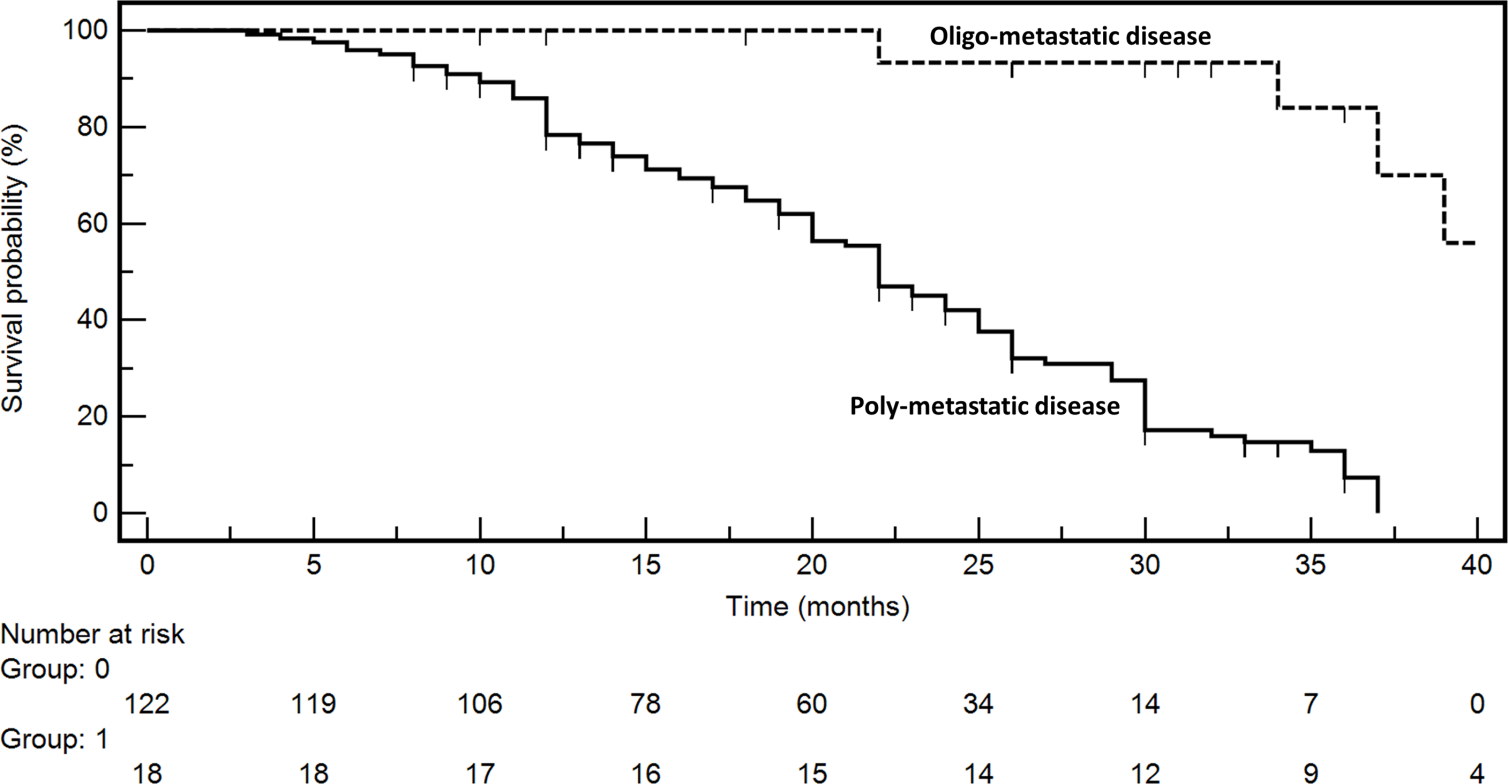
Kaplan–Meyer survival curves according to disease extent at diagnosis (oligometastatic vs. polymetastatic status).

The prognosis of oligometastatic CRC patients was evaluated in a consecutive clinical series of 140 metastatic CRC patients treated at the Istituto Nazionale Tumori (National Cancer Institute), “G. Pascale” Foundation from January 2018 to December 2021. Clinicopathological characteristics of the whole series are reported in **Table 1**. In multivariate analysis (**Table 2**), oligometastatic status was the only prognostic independent variable (HR: 0.08; 95% CI: 0.02–0.26; *p* < 0.001). The median survival in oligometastatic patients (18 patients) was not reached, while that of polymetastatic patients (122 patients) was 22.0 months (*p* < 0.0001 at Log-Rank test) (**Figure 1**). Six oligometastatic patients died of CRC progression. Nine out of 12 patients with genuine oligometastatic disease who were still alive at the last follow-up were phenotypically and genetically characterized (three patients refused to undergo further characterization for personal motivations). Their detailed characteristics are reported in **Table 3**. Interestingly, a genetic regression of *KRAS* was observed in three patients. Two patients (PAT2 and PAT5) had a double mutation in *KRAS* (p.G12V, p.G13D) in the primary tumor and a single mutation (p.G13D) in the metastatic one. PAT8 presented *KRAS* p.G12D mutated in the primary tumor and wild-type *KRAS* in the liver metastasis.

**Table 1:** Clinicopathological characteristics of metastatic CRC patients’ cohort.

Characteristic	No. (%)
**Age**	
<65	91 (65.0)
>65	49 (35.0)
**Gender**	
M	71 (50.7)
F	69 (49.3)
**Grading**	
G1	8 (5.7)
G2/G3	132 (94.2)
**Side of primary tumor**	
Left	73 (52.1)
Right	67 (47.9)
**pT^a^**	
pT1/pT2	11 (8.7)
pT3	87 (69.1)
pT4	28 (22.2)
**LN^a^ involvement**	
0	11 (8.7)
1–3	22 (17.5)
>3	93 (73.8)
**No. of metastatic sites**	
1	32 (22.9)
2	73 (52.1)
More than 2	35 (25.0)
**Metastatic tumor volume**	
¾3 cm^b^	10 (7.1)
>3–7 cm^b^	8 (5.7)
>7 cm	122 (87.1)
**Response to first-line chemotherapy**	
DC	116 (82.8)
No DC	24 (17.2)
***KRAS* status**	
Mutated	58 (41.4)
Wild-type	82 (58.6)

pT, pathological staging of primary tumor according to AJCC; pN, pathological staging of loco-regional lymph-node involvement according to AJCC; DC, disease control; F, female; LN, lymph nodes; M, male. ^a^The row sum does not correspond to the total number of patients because some of them did not receive surgical removal of the primary tumor. ^b^Oligometastatic disease.

**Table 2:** Multivariate analysis of clinical and molecular characteristics in the selected clinical cohort.

Covariate	Dichotomization	Median survivals	No. of events/patients	*p* at univariate	HR	95% CI	*p* at multivariate
**Age**	<65 vs. ≥65 years	25 vs. 22	63/91 vs. 35/49	0.39	1.09	0.71–1.68	0.67
**Gender**	M vs. F	24 vs. 24	52/71 vs. 46/69	0.51	1.18	0.78–1.78	0.41
**Side**	L vs. R	26 vs. 22	50/73 vs. 48/67	0.18	1.13	0.73–1.74	0.57
**Initial metastatic TB**	omCRC vs. pmCRC	NR vs. 22	94/122 vs. 4/18	<0.0001	0.08	0.02–0.26	<0.001
**Response to first-line CT**	DC vs. no DC	26 vs. 18	40/69 vs. 58/71	0.03	0.61	0.43–0.97	0.60
***KRAS***	Not mutated vs. mutated	26 vs. 22	56/82 vs. 42/58	0.35	1.07	0.70–1.62	0.74

CI, confidence interval; DC, disease control; F, female; HR, hazard ratio; L, left; M, male; NR, not reached; om, oligometastatic; pm, polymetastatic; R, right; TB, tumor burden.

**Table 3:** Clinicopathological characteristics and genetic concordance of *KRAS* status in the oligometastatic patients.

Patient ID	Colon PT *KRAS* status	MT *KRAS* status	Age (years)	Gender	BMI (kg/m^2^)	Histology and pathologic stage at diagnosis, grading	Primary tumor side	Synchronous or metachronous metastatic disease	Type of surgery for metastases	Last follow-up (December 2021)
PAT1	wt	Lung, wt	72	F	19.5	Adenocarcinoma, pT3pN1apV0 pR0, G2	Left	M	Lung left lower lobe wedge resection	Oligometastatic progression to the lung
PAT2	p.G12V, p.G13D	Lung, p.G13D	78	M	19.8	Adenocarcinoma, pT3pN0pV1 pR0, G2	Right	M	Lung left lower lobe wedge resection	Oligometastatic progression to the lung
PAT3	wt	Liver, wt	82	F	21.0	Adenocarcinoma, pT2pN1bpV0 pR0, G3	Right	M	Segment 5 and segment 6 pR0 metastasectomies	NED
PAT4	wt	Lung, wt	78	F	18.2	Adenocarcinoma, pT3pN1bpV1 pR0, G2	Right	M	Lung right lower lobe wedge resection	Polymetastatic progression to the lung
PAT5	p.G12V, p.G13D	Liver, p.G13D	61	M	19.0	Adenocarcinoma, pT2pN2bpV1 pR0, G2	Left	S	Segment 6 pR0 metastasectomy	NED
PAT6	wt	Liver, wt	27	F	18.9	Adenocarcinoma, pT3pN1bpV1 pR0, G2	Right	S	Segment 5 pR0 metastasectomy	NED
PAT7	wt	Liver, wt	52	M	21.2	Adenocarcinoma, pT2pN1apV1 pR0, G3	Left	M	Segment 8 pR0 metastasectomy	NED
PAT8	p.G12D	Lung, wt	60	M	19.9	Adenocarcinoma, pT3pN0bpV0 pR0, G2	Left	M	Lung right upper lobe wedge resection	NED
PAT9	wt	Liver, wt	47	F	20.5	Adenocarcinoma, pT3pN1apV1 pR0, G3	Left	S	Segment 8 pR0 metastasectomy	Oligometastatic progression to the liver

BMI, body mass index; G, grading; L, left; MT, metastatic tumor; PAT, patient; PT, primary tumor; pR0, no residual disease at microscopic examination after surgery; wt, wild-type.

### Tumor Immune Microenvironment Characterization

**Figure 2:**
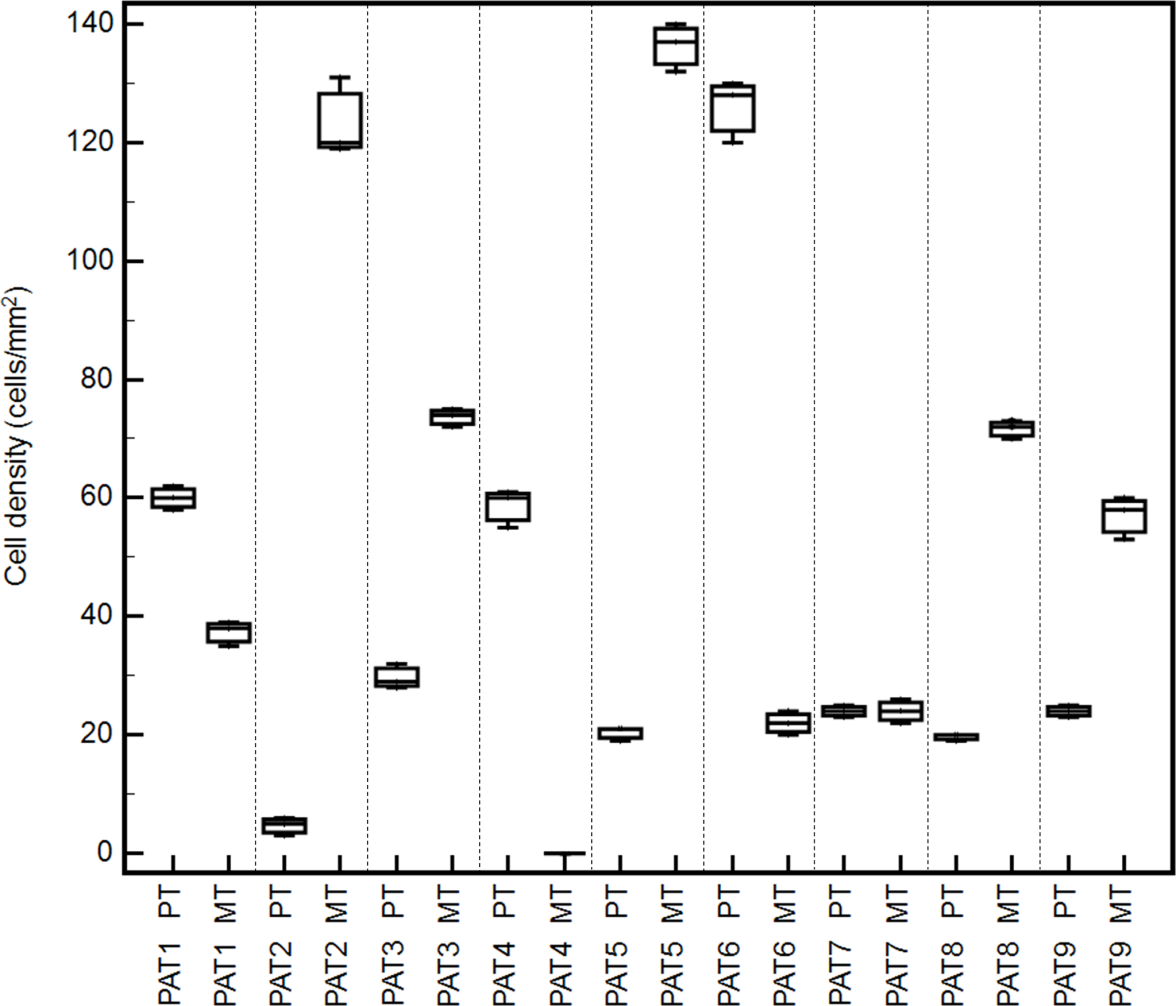
Box-and-whisker graphs of GrzB^+^ cell densities into tumor cores of different oligometastatic patients (PAT1-9) in matched metastatic (MT) and primary tumors (PT).

**Figure 3:**
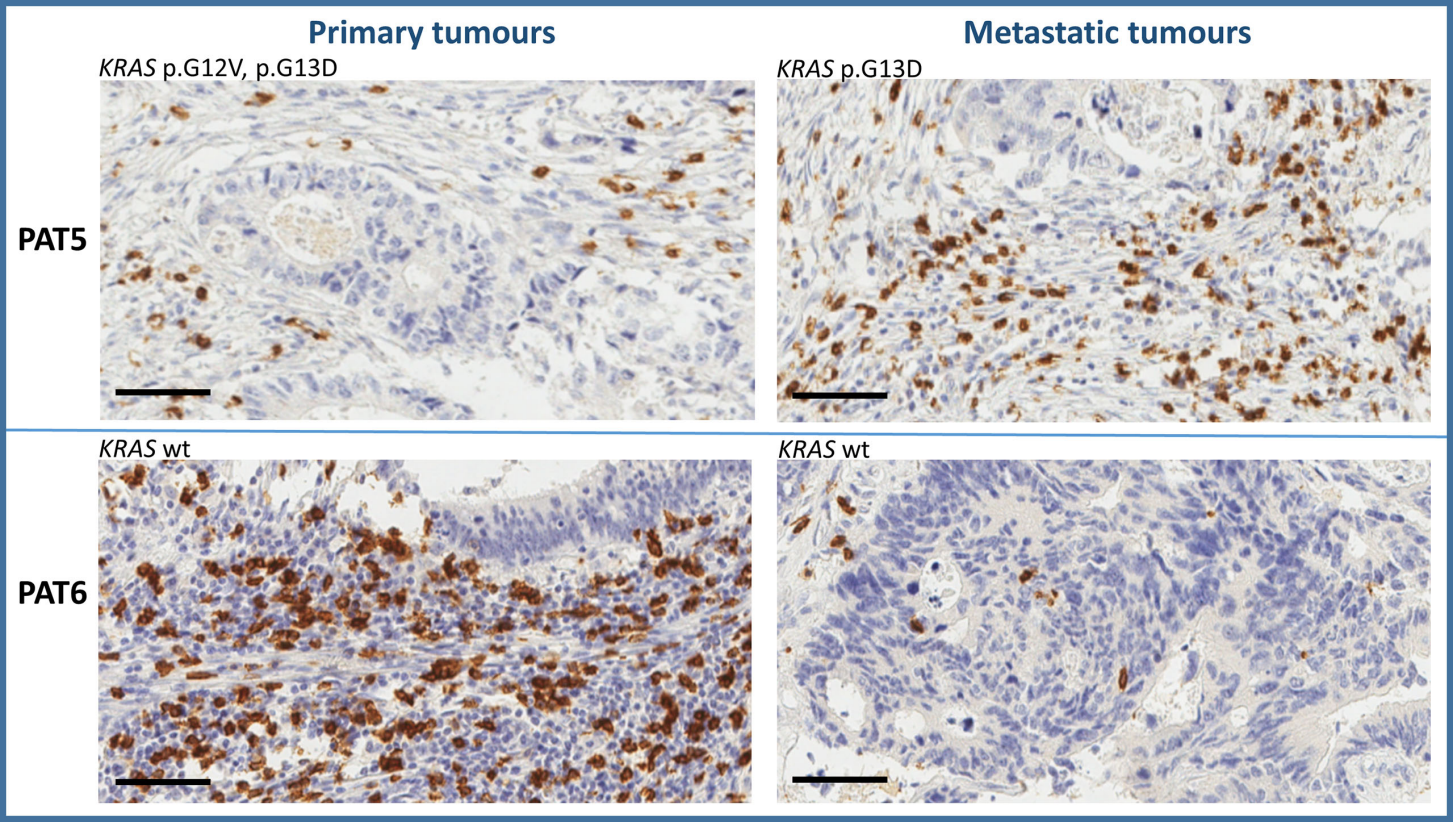
Representative immunohistochemistry of GrzB^+^ cells in tumor cores of PAT5 and PAT6 (bar = 100 µm).

Tumor immune microenvironment (TIME) has a strong influence on tumor progression, and TILs are able to shape the clonal heterogeneity of malignant cells in space and time. Therefore, to explore any relevant relationship between the oligometastatic status of the selected patients and the immunological microenvironment, CD3^+^, CD8^+^, FoxP3^+^, and GrzB^+^ cells were examined in the primary and metastatic tissues by IHC. Cell densities in means ± 2SD are reported in **Table 4**. As expected, a great variability of cell densities inter- and intrapatient (primary vs. metastatic tumor) was recorded. Different densities of GrzB^+^ cells (fully differentiated and activated lymphocytes with lytic properties infiltrating the tumor core) into the tumor cores of both primary and metastatic tissues are shown in **Figure 2**. PAT6 had the highest number of GrzB^+^ cells in the primary tumor (120 ± 13.3 cells/mm (2)), while PAT2 (120 ± 11.2 cells/mm (2)) and PAT5 (132 ± 12.2 cells/mm^2^) had the most in the metastatic tumors. Representative IHCs of GrzB^+^ cells in tumor cores of PAT5 and PAT6 are shown in **Figure 3**.

**Table 4:** Distribution of T-cell subset densities (cells/mm^2^) in primary and oligometastatic tumors.

	Primary tumors								Metastatic tumors							
	CD3^+^		CD8^+^		Foxp3		GrzB		CD3^+^		CD8^+^		Foxp3		GrzB	
	TC	IM	TC	IM	TC	IM	TC	IM	TC	IM	TC	IM	TC	IM	TC	IM
**PAT1**	170 ± 12.0	460 ± 20.5	80 ± 5.5	170 ± 12.5	10 ± 1.4	30 ± 4.2	60 ± 3.7	20 ± 3.1	340 ± 10.2	1,350 ± 25.6	580 ± 20.3	2,140 ± 30.9	0 ± 0.0	20 ± 1.6	35 ± 3.5	50 ± 1.1
**PAT2**	720 ± 16.5	230 ± 10.0	30 ± 2.4	95 ± 11.5	0 ± 0.0	0 ± 0.8	5 ± 0.6	0 ± 1.2	140 ± 7.2	625 ± 20.1	25 ± 2.2	85 ± 5.5	0 ± 0.0	5 ± 0.6	120 ± 11.2	100 ± 9.8
PAT3^a^	600 ± 15.5	2,000 ± 21.2	50 ± 5.5	150 ± 11.1	67 ± 5.4	40 ± 4.2	32 ± 2.7	45 ± 3.3	200 ± 9.9	1,500 ± 24.9	50 ± 2.3	120 ± 8.7	20 ± 2.1	40 ± 3.6	74 ± 6.8	30 ± 2.2
**PAT4**	160 ± 9.5	365 ± 13.1	15 ± 1.2	75 ± 8.4	0 ± 0.0	0 ± 0.0	60 ± 5.1	115 ± 9.6	220 ± 9.9	1,015 ± 23.8	140 ± 10.2	120 ± 8.7	5 ± 0.7	10 ± 1.3	0 ± 0.0	0 ± 0.0
**PAT5**	770 ± 13.3	507 ± 21.2	62 ± 6.6	726 ± 21.5	52 ± 4.3	25 ± 4.5	21 ± 1.3	29 ± 2.9	201 ± 11.2	201 ± 2.3	132 ± 11.3	120 ± 11.6	10 ± 0.9	30 ± 2.9	132 ± 12.2	60 ± 9.6
**PAT6**	820 ± 19.7	1,500 ± 17.2	600 ± 26.2	500 ± 18.4	30 ± 3.3	20 ± 2.9	120 ± 13.3	110 ± 13.9	130 ± 8.2	1,610 ± 19.8	30 ± 2.8	60 ± 6.3	11 ± 0.7	15 ± 1.4	24 ± 2.6	35 ± 2.7
**PAT7**	2,000 ± 23.4	1,040 ± 16.1	100 ± 7.4	400 ± 12.3	30 ± 4.2	30 ± 3.3	25 ± 1.6	42 ± 2.9	310 ± 12.1	3,000 ± 20.7	100 ± 10.3	530 ± 21.6	28 ± 1.7	30 ± 2.6	26 ± 2.4	42 ± 3.3
**PAT8**	245 ± 7.0	865 ± 11.5	144 ± 11.6	166 ± 13.2	0 ± 0.0	7 ± 1.9	20 ± 0.8	0 ± 0.0	680 ± 15.3	2,020 ± 32.4	80 ± 5.8	650 ± 18.7	0 ± 0.0	6 ± 1.3	72 ± 6.4	30 ± 3.2
**PAT9**	400 ± 9.5	500 ± 23.2	80 ± 6.2	200 ± 8.4	40 ± 3.3	40 ± 3.9	25 ± 2.1	30 ± 2.6	530 ± 19.2	2,015 ± 23.8	78 ± 6.8	100 ± 8.8	10 ± 0.7	10 ± 1.4	58 ± 3.6	45 ± 4.8

Foxp3, forkhead box P3; GrzB, granzyme B; IM, invasive margins; MT, metastatic tumor; PT, primary tumor; TC, tumor core.

a This patient had two liver lesions both wtKRAS (cell subsets of the two lesions were comparable).

### Peripheral T and NK Cells from Oligometastatic CRC Patients Differently Target CRC Cell Lines Bearing Specific *KRAS* Mutations

**Figure 4:**
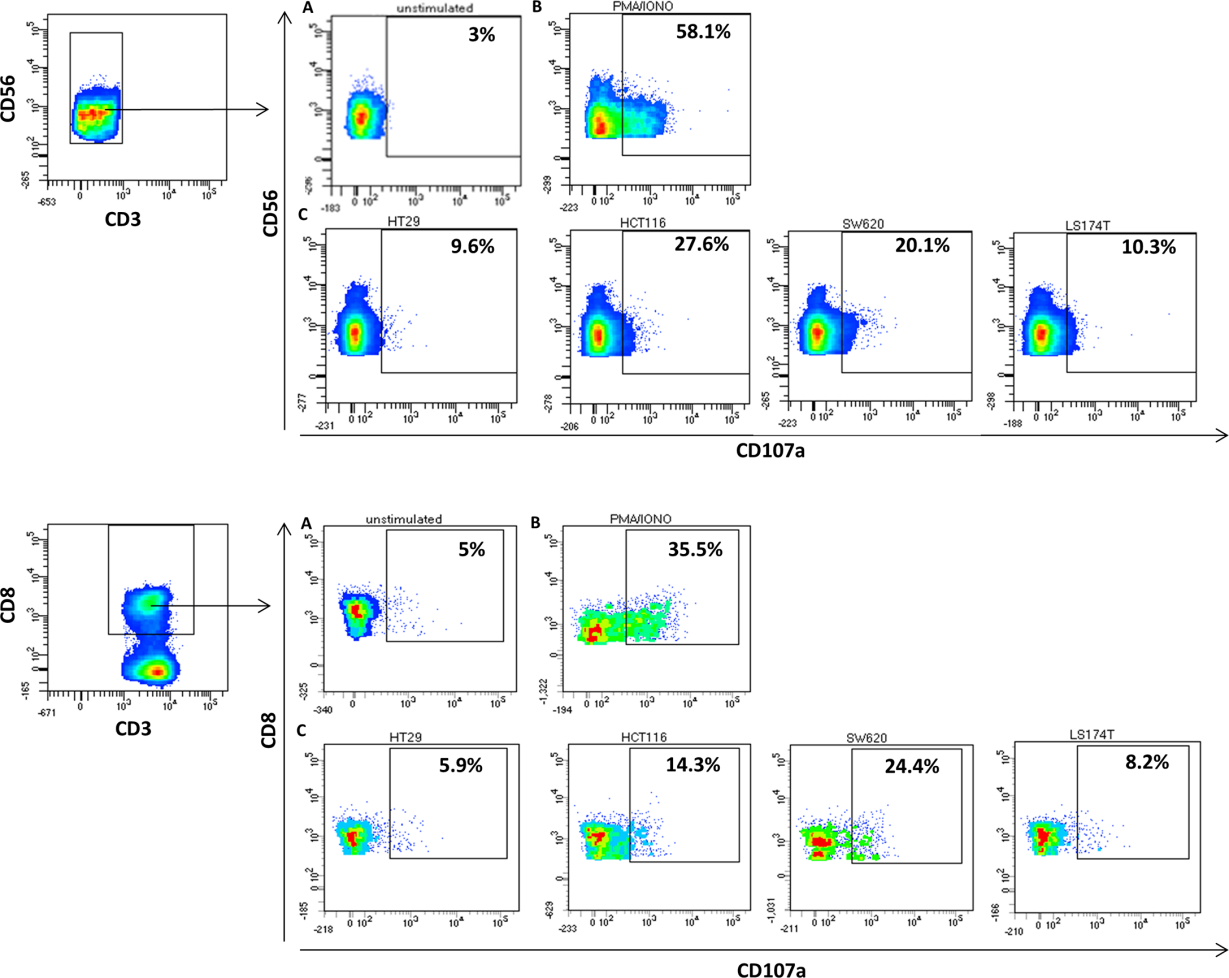
Differential cytotoxic activity in colon cancer oligometastatic patients (representative CD107a degranulation assay). Upper panel, NK-purified cells were gated based on (CD56^+^CD3^−^) **(A)** spontaneous, **(B)** PMA/IONO-induced, and **(C)** human colon cancer cell line-induced NK degranulation. Lower panel, T cells were gated based on (CD8^+^CD3^+^) **(A)** spontaneous, **(B)** PMA/IONO-induced, and **(C)** human colon cancer cell line-induced CD8^+^ T-cell degranulation.

**Figure 5:**
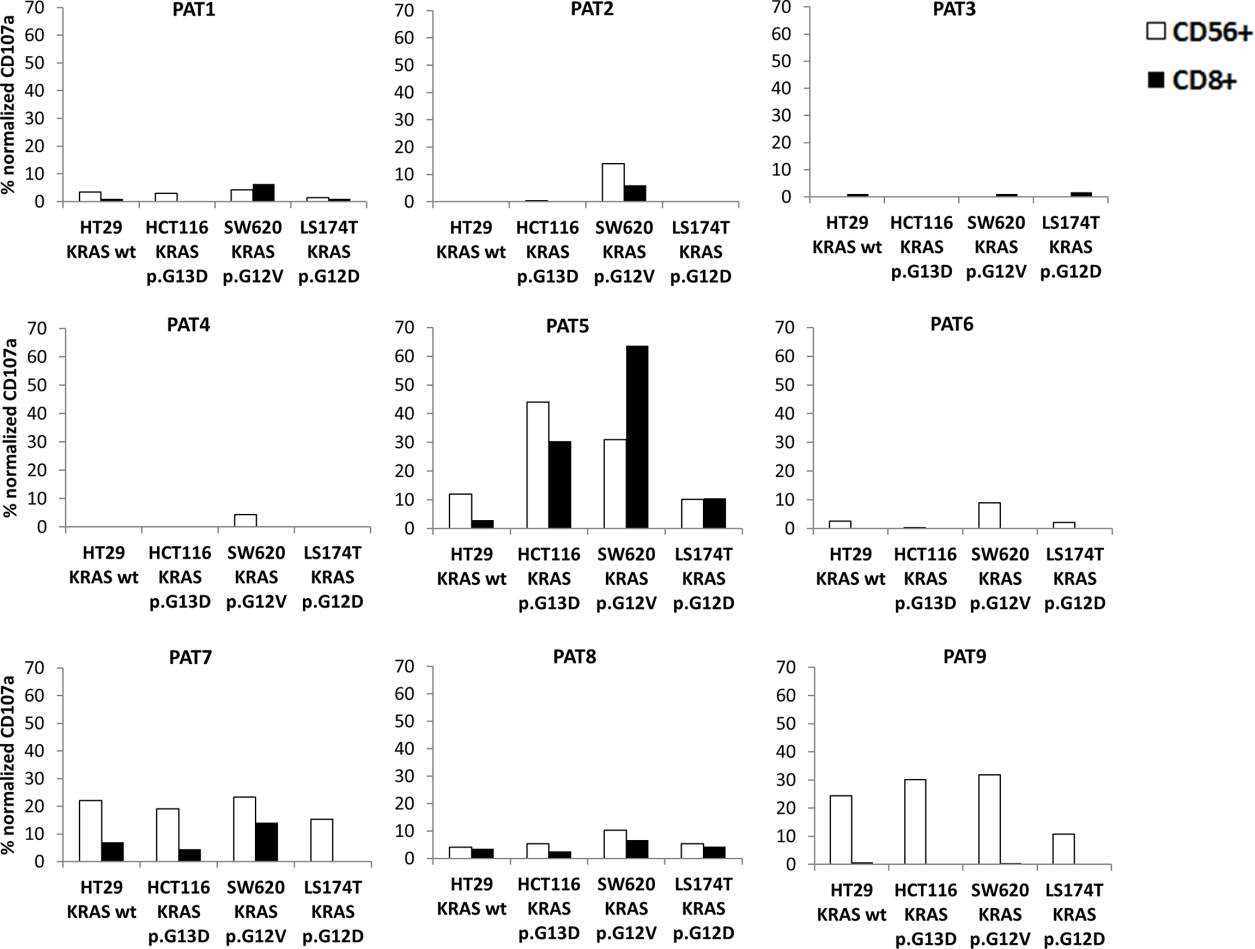
Cytotoxicity of peripheral lymphocytes from oligometastatic patients (PAT1-9) against different CRC target cells was evaluated through CD107a externalization (see *Methods*) on CD3^+^/CD8^+^ from NK-depleted PBMCs (black column) and CD3^−^/CD56^+^ NK cells (white column) (representative results of three experiments). Targets were HT29 (KRAS wild-type), HCT116 (KRAS p.G13D), SW620 (KRAS p.G12V), and LS174T (KRAS p.G12D). Normalized CD107a was the percentage of CD107a^+^ cells subtracted from spontaneous degranulation/PMA-IONO-induced degranulation (positive control).

Since regression of *KRAS* mutations has been reported in oligometastatic CRC patients, we explored NKs and CD8s from peripheral blood of oligometastatic patients for cytotoxic activity against CRC cells through CD107a-externalization (lysosomal protein LAMP-1-based degranulation assay) (**Figure 4**). The cytotoxic response against target cells is reported in **Figure 5**. It was composite and quantitatively heterogeneous among different patients. Notably, PAT2 and PAT5 had lymphocytes with the highest T cell-mediated cytotoxic activity against SW620 cells (p.G12V mutated *KRAS* CRC cells), which was consistent with the observed mutational regression (p.G12V/p.G13D inprimary→p.G13D in metastatic tumor).

### HLA Characterization of Selected Patients

The hypothesis that T lymphocytes from oligometastatic patients had the ability to recognize and efficiently kill specific mutated tumor cells was generated from the observation of PAT2 and PAT5 target killing patterns. Since HLA proteins are crucial in binding heterogeneous tumor antigens and driving T-cell immune-mediated recognition and elimination, patients’ HLA haplotype was determined (**Table 5**). Interestingly, 6 out of 9 patients expressed the HLA-C7 allele (see ***Discussion***). Matching between HLA haplotypes of patients and tumor target cells is reported in **Table 6**. Notably, both SW620 and HCT116 cell lines expressed HLA-C7 alleles.

**Table 5:** HLA haplotypes of oligometastatic patients.

Patients	Locus	Alleles	
**PAT1**	HLA-A^*^	01:01	03:01
	HLA-B^*^	35:08	53:01
	HLA-C^*^	04:01	–
	HLA-DRB1^*^	10:01	11:01
	HLA-DQB1^*^	03:01	05:01
	HLA-DPB1^*^	10:01P	14:01P
**PAT2**	HLA-A^*^	03:01	30:04
	HLA-B^*^	08:01	38:01
	HLA-C^*^	07:01	12:03
	HLA-DRB1^*^	01:02	03:01
	HLA-DQB1^*^	02:01	05:01
	HLA-DPB1^*^	03:01P	13:01P
**PAT3**	HLA-A^*^	02:17	03:01
	HLA-B^*^	15:17	35:01
	HLA-C^*^	04:01	07:01
	HLA-DRB1^*^	13:02	–
	HLA-DQB1^*^	05:02	06:03
	HLA-DPB1^*^	02:01P	–
**PAT4**	HLA-A^*^	02:01	33:01
	HLA-B^*^	07:02	14:02
	HLA-C^*^	07:02	08:02
	HLA-DRB1^*^	01:02	15:01
	HLA-DQB1^*^	05:01	06:02
	HLA-DPB1^*^	03:01P	14:01P
**PAT5**	HLA-A^*^	02:01	–
	HLA-B^*^	18:01	51:01
	HLA-C^*^	07:01	15:02
	HLA-DRB1^*^	04:03	11:01
	HLA-DQB1^*^	03:01	–
	HLA-DPB1^*^	02:01P	04:01P
**PAT6**	HLA-A^*^	23:01P	24:02
	HLA-B^*^	35:02	49:01
	HLA-C^*^	04:01	07:01
	HLA-DRB1^*^	11:04	–
	HLA-DQB1^*^	03:01	–
	HLA-DPB1^*^	02:01P	04:02P
**PAT7**	HLA-A^*^	02:01	03:01
	HLA-B^*^	07:05P	35:01
	HLA-C^*^	04:01	15:05
	HLA-DRB1^*^	13:01	16:01
	HLA-DQB1^*^	05:02	06:03
	HLA-DPB1^*^	04:01P	–
**PAT8**	HLA-A^*^	24:02	–
	HLA-B^*^	35:02	37:01
	HLA-C^*^	04:01	06:02
	HLA-DRB1^*^	10:01	11:04
	HLA-DQB1^*^	03:01	05:01
	HLA-DPB1^*^	03:01P	04:01P
**PAT9**	HLA-A^*^	24:02	–
	HLA-B^*^	18:01	51:01
	HLA-C^*^	07:01	15:02
	HLA-DRB1^*^	04:03	11:01
	HLA-DQB1^*^	03:01	–
	HLA-DPB1^*^	02:01P	04:01P

**Table 6:** Matching of HLA classes I and II between analyzed patients and CRC cell lines (red: HLA class I matching; blue: HLA class II matching).

		HT29							HCT116							SW620							LS174T
		A	B	C	DRB1	DQB1	DPB1		A	B	C	DRB1	DQB1	DPB1		A	B	C	DRB1	DQB1	DPB1		A
**PAT1**	**A**																						
	**B**																						
	**C**																						
	**DRB1**																						
	**DQB1**																						
	**DPB1**																						
																			
**PAT2**	**A**																						
	**B**																						
	**C**																						
	**DRB1**																						
	**DQB1**																						
	**DPB1**																						
																			
**PAT3**	**A**																						
	**B**																						
	**C**																						
	**DRB1**																						
	**DQB1**																						
	**DPB1**																						
																			
**PAT4**	**A**																						
	**B**																						
	**C**																						
	**DRB1**																						
	**DQB1**																						
	**DPB1**																						
																			
**PAT5**	**A**																						
	**B**																						
	**C**																						
	**DRB1**																						
	**DQB1**																						
	**DPB1**																						
																			
**PAT6**	**A**																						
	**B**																						
	**C**																						
	**DRB1**																						
	**DQB1**																						
	**DPB1**																						
																			
**PAT7**	**A**																						
	**B**																						
	**C**																						
	**DRB1**																						
	**DQB1**																						
	**DPB1**																						
																			
**PAT8**	**A**																						
	**B**																						
	**C**																						
	**DRB1**																						
	**DQB1**																						
	**DPB1**																						
																			
**PAT9**	**A**																						
	**B**																						
	**C**																						
	**DRB1**																						
	**DQB1**																						
	**DPB1**																						

## Discussion

There is increasing attention to the difference between oligo- and polymetastatic cancers in CRC. The genuine oligometastatic setting has a long-term course with indolent and safely controllable disease. Some patients subjected to R0 surgical resection of the primary tumor and the presence of oligometastases do not progress rapidly, differently from polymetastatic patients. Unfortunately, the biological and molecular determinants of these so divergent clinical behaviors (oligo- vs. polymetastatic cancer) remain unexplored and unknown. We previously reported that genetic loss of key-driver gene mutations (“genetic regression”) might represent a new hallmark of the oligometastatic disease (2). Moreover, a recent study was performed on tumor and matched metastatic tissues collected from 16 patients with CRC and subjected to whole-exome sequencing and RNA sequencing. The authors found recurrent mutations with subclonal changing patterns in different genes, including *KRAS*, *SYNE1*, *CACNA1H*, *PCLO*, *FBXL2*, and *DNAH11*, giving evidence of a potential mechanism of tumor cell immune escape by analyzing HLA-related clonal neoantigens and immune cell components in CRC liver metastases (15).

Based on that, we provide further evidence that peripheral CD3^+^/CD8^+^ lymphocytes of oligometastatic CRC patients recognize and eliminate differentially *KRAS*-mutated CRC cells. In fact, a genetic regression of *KRAS* was observed in three patients: PAT2, PAT5, and PAT8. PAT2 and PAT5 presented a double mutation in *KRAS* (p.G12V, p.G13D) in the primary tumor and only a single mutation (p.G13D) in the metastasis. PAT8 presented p.G12D-mutated *KRAS* in primary tumor and wild-type *KRAS* in the liver metastasis; nevertheless, he presented neither a specific pattern nor a relevant cytotoxic activity against tumor target cells, with degranulation rates ranging from 5% to 10% for both NK and T cells. The last data can be attributable to heterogeneous, unknown underlying factors orchestrating the immune responses and tumor progression relationships. *KRAS* double mutations are described in the literature and, although rare events, they can represent an interesting clinical model to study tumor genetic evolution (16–19).

In degranulation assays, both PAT2 and PAT5 had CD3^+^/CD8^+^-dependent cytotoxicity against SW620 (p.G12V *KRAS*); PAT5 also displayed high recognition of the HCT116 cell line (p.G13D *KRAS*). Interestingly, genetic characterization of resected metastases from patients PAT2 and PAT5 demonstrated loss of *KRAS* p.G12V-mutated neoplastic progeny. At the last follow-up (December 2021), PAT2 had two new lung nodules at radiologic restaging while PAT5 was disease free. This could be related to the ability of PAT5 to destroy the residual *KRAS* p.G13D neoplastic clones. Thus, the patients’ cytotoxic properties seemed consistent with the mutational and clinical course of the neoplastic progeny. The mutated *KRAS* G12D and G12V have been reported as recurrent neoantigens in CRC, and this supports the hypothesis about the role of immune-mediated clonal selection in the above patients (20), which deserves further studies to be definitely addressed.

The study of TIME suggests that lymphocytes are involved in shaping, at both loco-regional and systemic levels, the clonal heterogeneity of CRC cells of oligometastatic patients with genetic regression. In fact, PAT2 and PAT5 had a high density of GrzB^+^ cells (fully differentiated and activated lymphocytes (21, 22)) in the metastatic tissues.

A HLA haplotype characterization was conducted considering the crucial role of these proteins in mediating CD3^+^/CD8^+^ recognition of tumor cells. Noteworthy, 6 out of 9 patients (66.7%) presented an HLA-C7 allele (matching both SW620 and HCT116 cell lines). The HLA system is a group of highly polymorphic membrane-bound proteins involved in presenting processed tumor antigens and stimulating T-cell responses through binding to T-cell receptor (TCR). The possible combinations of different HLA loci on an HLA haplotype are enormous (23). According to the National Marrow Donor Program (NMDP, http://bioinformatics.nmdp.org/) and IPD-IMGT/HLA databases (http://www.ebi.ac.uk/imgt/hla), HLA-C7 is detected in about 17% of Caucasians. This provides evidence that HLA-C7 in oligometastatic CRC patients is more frequent than expected by chance. However, considering the small number of analyzed patients (about 5% of metastatic CRC patients have a genuine and true oligometastatic disease), such a hypothetic and unexpected result deserves to be confirmed in larger clinical series. Further studies are needed to address any interesting relationships between oligometastatic status, specific *KRAS* mutations, and HLA haplotypes.

Our study has some limitations, which deserve to be revealed and discussed. First, the nonspecific/nonantigen-related nature of the immune assays does not allow us to identify eventual immunogenic KRAS-derived epitopes. Second, the small sample size related to the high selection of the tested cohort (less than 5% of metastatic CRC patients have a genuine and good prognosis of oligometastatic disease). Third, we did not perform a time-course of patients’ lymphocyte characterizations although the reported experiments gave homogeneous results and are representative of three of them. The blood was recovered at different times considering the distance from resection of oligometastases. Fourth, patients 1, 4, 5, 6, 7, and 9 underwent standard adjuvant chemotherapy based on fluoropyrimidines and oxaliplatin after primary tumor resection. In this case, particularly in patients developing metachronous metastatic disease, we cannot exclude the chemotherapy effect in shaping the clonal heterogeneity of subsequent metastases. Finally, the biological diversity of CRC cell lines (different haplotypes, costimulatory molecules, cytokines, etc.) could be associated with different background recognitions. However, all these factors reasonably account for physiologic heterogeneity of patients’ data, paradoxically reinforcing the strength of specific results in some of them and, thus, the generation of hypotheses.

The secretion of lytic granules from NK and CD8^+^ lymphocytes involves the fusion of the granule membrane with the cytoplasmic membrane of the immune effector cell, resulting in the surface exposure of lysosomal-associated proteins that are typically present on the lipid bilayer surrounding lytic granules, such as CD107a. Therefore, membrane expression of CD107a constitutes a marker of immune cell activation and cytotoxic degranulation. Nevertheless, this assay may not be ideal in the setting of patient-derived NK and CD8^+^ tests toward human colon cancer cells. Although HLA-typing and matching for cell lines is reported, PAT5 highly matched the HLA haplotype of HCT116 and SW620 human colon cancer cells, alloreactivity could justify some effects (**Supplementary File S1** reports the HLA haplotypes of tested cell lines). However, comparing CD107a activity in patient-derived cells to cell lines with HLA and KRAS mutations suggests that CD107a activity is also affected by KRAS mutation status.

In conclusion, activated and reactive lymphocytes can be isolated from the peripheral blood of oligometastatic CRC patients with *KRAS* regression. The hypothesis of the active elimination of mutated neoplastic clones, although requiring further and much stronger data, implies specific and innovative monitoring and therapeutic strategies.

## Data Availability Statement

The data presented in the study are deposited in the Zenodo repository, accession number https://zenodo.org/record/6350253#.Yr1kO3ZByUl.

## Ethics Statement

Ethical review and approval were not required for retrospective studies. Authorization from the Scientific Directorate in the context of an ongoing retrospective no-profit study on long-term survivors in CRC for recovering additional blood from oligometastatic patients was obtained.

## Author Contributions

Conceptualization: AO, MiC, and GN. Methodology: AO, RPd’A., and MiC. Software: GSc, GSa, and MS. Validation: FT and GSc. Formal analysis: AO, GN, and FP. Investigations, flow-cytometry: MN, SS, and AT. Surgery: FI and AB. Investigations, genetic assessments: RPd’A, MaC, AL, PG, MI, SZ, NP, and GSa. Investigations, radiologic assessments: VG. Resources: AO and GN. Data curation: AO, MiC, GN, SS, and FP. Writing–original draft preparation: AO, GN, and MS. Writing—review and editing: FP, AL, and MiC. Supervision: GN. All authors have read and agreed to the published version of the manuscript.

## Funding

We acknowledge the “Lega Italiana per la Lotta contro I Tumori (LILT)‐Sezione di Napoli” for collaboration and financial support. We also greatly thank Mrs. Antonietta Nacca (a private citizen) for her free and unconditional financial support.

## Conflict of Interest

GSa, MI, MaC, and NP are employed by AMES, Centro Polidiagnostico Strumentale srl, Naples, Italy.

The remaining authors declare that the research was conducted in the absence of any commercial or financial relationships that could be construed as a potential conflict of interest.

## Publisher’s Note

All claims expressed in this article are solely those of the authors and do not necessarily represent those of their affiliated organizations, or those of the publisher, the editors and the reviewers. Any product that may be evaluated in this article, or claim that may be made by its manufacturer, is not guaranteed or endorsed by the publisher.
