# Supplementary figures and images for: Characterization of KRAS Mutational Regression in Oligometastatic Patients
Source: Front Immunol. 2022 Jul 22;13:898561. doi: 10.3389/fimmu.2022.898561 (PMC9354788; doi:10.3389/fimmu.2022.898561)

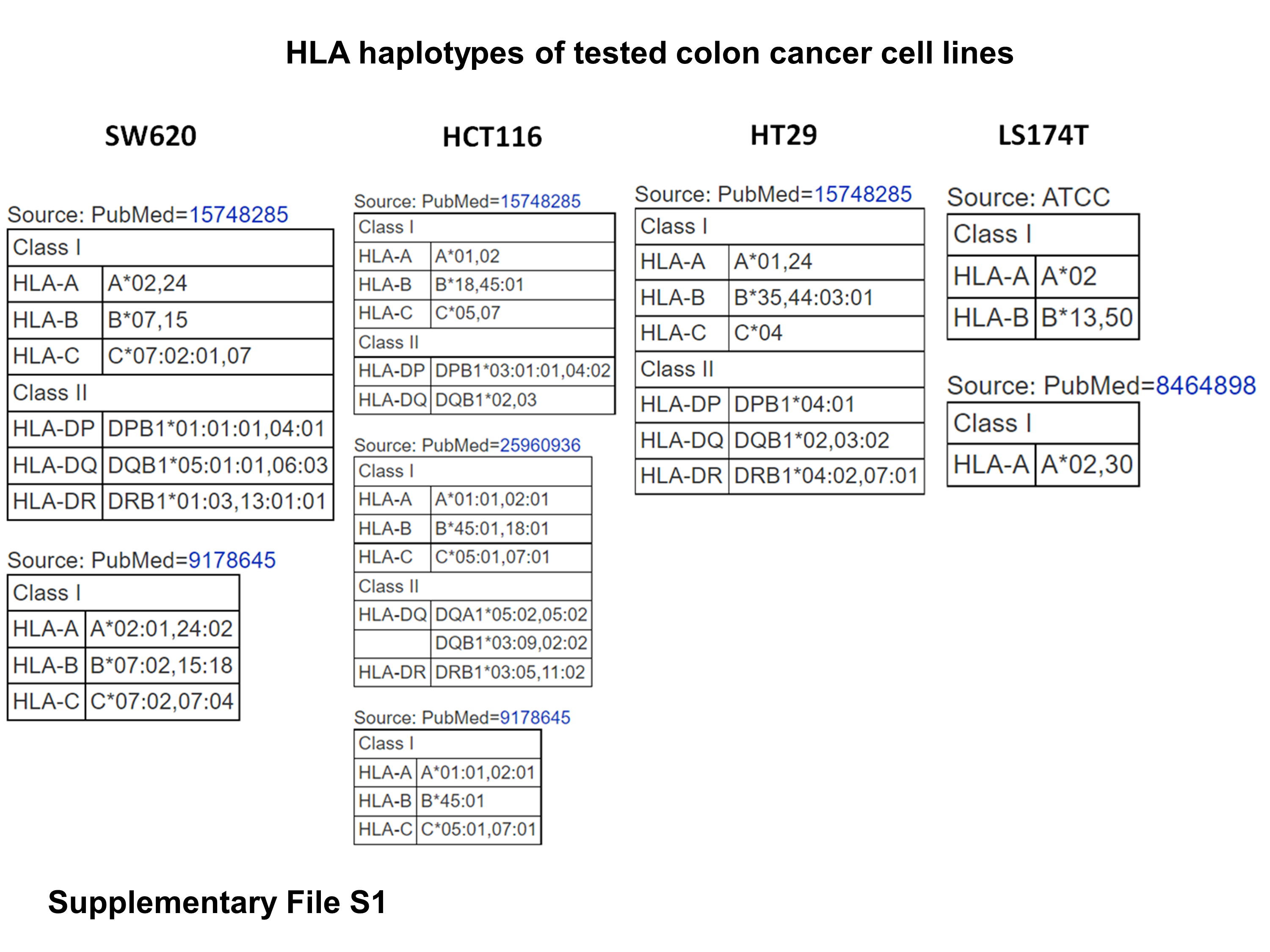

Supplement: Supplementary file 1 [file Image_1.tif]
